# Combination drug therapy reduces iron accumulation and microglia-mediated pathologies in neonatal intraventricular hemorrhage: a biochemical and transcriptomic analysis

**DOI:** 10.3389/fncel.2026.1812529

**Published:** 2026-05-25

**Authors:** Vanessa Castro Diaz, Michelle Sunshine, Furong Hu, Sohan Shah, Weihua Huang, Carl I. Thompson, Michael S. Wolin, Selvakumar Subbian, Edmund F. LaGamma, Govindaiah Vinukonda

**Affiliations:** 1 The Regional Neonatal Center, Maria Fareri Children’s Hospital at Westchester Medical Center, Division of Pediatric-Newborn Medicine, New York Medical College, Valhalla, NY, United States; 2 Department of Pediatrics, New York Medical College, Valhalla, NY, United States; 3 Department of Pathology and Laboratory Medicine, Brody School of Medicine at East Carolina University, Grenville, NC, United States; 4 Department of Cell and Molecular Physiology, New York Medical College, Valhalla, NY, United States; 5 Public Health Research Institute, New Jersey Medical School, Rutgers, The State University of New Jersey, Newark, NJ, United States

**Keywords:** brain injury, corona radiata, corpus callosum, deferoxamine, ferroptosis, gene networks, germinal matrix, intraventricular hemorrhage

## Abstract

This study describes the distribution of non-reactive brain-resident microglia densely populated along the borders of the lateral ventricles and choroid plexus in premature rabbit pups during early forebrain development. Following intraventricular hemorrhage (IVH) injury, activated microglia expand by proliferation, and migrate deeper into parenchymal regions. During this process, activated microglia exhibit a disproportionate elevation of the proinflammatory microglia phenotype (M1 nomenclature) from the total IBA-1^+^ microglia cell population along with tissue iron accumulation; this shift was reduced by sulforaphane (SFN; Nrf2-antioxidant response element [ARE] activator of anti-inflammatory pathways) plus deferoxamine (DFN; iron chelator) treatment. A separate DFN monotherapy transcriptome analysis identified over expression of pro-inflammatory calcium-binding proteins S100A8 and S100A12 (intracellular damage signals), as well as chemokines CXCL8 and CXCL10 by microglia and other cells, along with upregulated ferroptosis interactive network genes in IVH including: HMOX1, CTSB, FTL, PRM2, LPCAT1, and CDK1. Importantly, the expression of multiple key genes involved in iron metabolism and transport function included: ACSL4, TFRC, SLC7A11 and ABCA4 which were all downregulated in IVH and this trend was reversed after DFN treatment. Taken together, in the developing postnatal brain, the combination treatment of SFN-DFN mitigated M1 infiltration, reduced iron deposition in the tissue and in the CSF, suppressed the magnitude of inflammation and reduced cell death after IVH. Moreover, DFN monotreatment reversed most dysregulated genes in inflammation and iron homeostasis networks, revealing potential molecular targets for additional pharmacologic interventions after IVH. We speculate that reducing the toxic microcellular environment will attenuate injurious inflammatory responses and improve recovery of the trajectory toward normal brain development.

## 1 Introduction

Intraventricular hemorrhage (IVH) is a common problem of premature newborns and is associated with white matter injury (WMI), long-term neuromotor delay and cognitive disabilities (Volpe, 2015). Hemorrhagic extravasation of red blood cells (RBC) hemolyze and locally release toxic byproducts including free hemoglobin (heme and globin), free iron, and iron-induced free radicals contributing to an inflammatory cytokine influx and additional collateral injury (Bulbake et al., 2019). Despite advances in perinatal care over the past 30 years, a recent meta-analyses showed that the overall prevalence of IVH in extremely premature neonates (≤ 28 weeks gestational age) is unchanged at approximately 34% (Lai et al., 2022; Nagy et al., 2025; Nagy et al., 2025). However, at the core of this phenomenon is the fact that in the earliest gestational ages, the absence of clearly effective preventative measures for IVH that directly address the structural vulnerabilities of the maturing brain, remains enigmatic (Ballabh, 2010). Moreover, no definitive therapeutic intervention exists for IVH, and clinical management is primarily supportive care. Since prevention has had limited success, we reasoned that addressing mechanisms of injury initiated at early phases of the temporal progression after IVH would likely mitigate the extent of subsequent or derivative inflammatory processes enhancing the potential for recovery of normal brain development. We speculate that preferential conditions should extend survival of “*living cellular therapy*” to better harness the evolutionary leverage of exogenous stem cells that enhance recovery from brain injury (Vinukonda and La Gamma, 2022).

Mechanistically, IVH originates within the subependymal germinal matrix (GM), a highly vascularized and cellular brain region prominent between 22 and 32 weeks of gestation in humans (Del Bigio, 2011). The GM is a critical neurogenic niche that produces migrating neuro-glial precursor cells to form all layers of the adult brain. Hemorrhagic disruption of the GM compromises the ependymal lining allowing extension into the lateral ventricles, where severe bleeding encompasses both the sub-ventricular (SVZ) and ventricular zones (VZ) of the brain parenchyma (Purohit et al., 2021; Finkel et al., 2023). The presence of extravasated blood within these areas initiates a temporally predictable cascade of pathophysiologic events: reduced nutrient delivery due to ruptured blood vessels, extravasated red blood cells (RBCs) causing a secondary mass compression effect, then hemolysis and breakdown of hemoglobin into heme, globin and free iron. Further, the RBC lysis resulting in free hemoglobin and iron catalyzes the formation of highly toxic free radicals triggering collateral release of cytotoxic inflammatory byproducts from activated microglia that serve a dual role as essential modulators of injury and recovery. The injury progression with excessive free radical production results in a secondary late phase extension of the injury involving inflammation and apoptotic cell death. Derivative of these events is a reduction in normal growth factors that ultimately, impedes the recovery to normal brain development (Gram et al., 2014; Bozza and Jeney, 2020; Dawes, 2022; Shao et al., 2022).

After injury resident non-reactive, microglia undergo morphological transformation into an activated phenotype, characterized by hypertrophic or amoeboid morphology with reduced process complexity. The classically activated M1 nomenclature phenotype secretes pro-inflammatory cytokines and reactive oxygen species (ROS), while the alternatively activated M2 phenotype, facilitates phagocytosis, anti-inflammatory, and tissue repair processes (Gomes-Leal, 2012). Furthermore, microglia participate in release of damage associated molecular pattern molecules (DAMPs) due to IVH pathology, which further accelerate inflammation.

The pivotal contribution of microglia to IVH pathology is not surprising as they constitute up to 10% of the total cell population in the rabbit brain and up to 16.6% in the human brain, depending on the anatomical region (Lawson et al., 1992). Moreover, in the developing human brain, resident microglia arrive from erythromyeloid precursors in the yolk sac early in the first trimester and are low in density (Choi, 1981; Fujimoto et al., 1989; Andjelkovic et al., 1998; Gao et al., 2023). By 24 weeks gestation, large numbers of microglia with short process can be identified in the germinal matrix and subventricular zone (Hutchins et al., 1990). Subsequently, microglia mature into a terminally differentiated ramified morphology and further accumulate in density in all anatomical areas until after birth (Perry et al., 1985, Lawson et al., 1990, Kostovic and Judas, 2002, Schmid et al., 2009). Once morphology and migration are established, microglial populations are maintained via a self-renewal process throughout life with a median turnover rate of 28% annually (~ 0.08% per day) and an estimated lifespan of 4.2 years in the human cortex (Reu et al., 2017). We hypothesized that by attenuating early-stage microglia activation after IVH injury, we can reduce the overall magnitude of the prolonged inflammatory state and improve recovery.

In this report, to gain insights into the complexity of these processes and perhaps those relevant but not yet fully recognized, we took advantage of RNAseq technology to elucidate changes in gene expression and associated signaling pathways in the developing brain after IVH. We attempted to redirect the pathophysiology of injury by removing free iron with deferoxamine (DFN) treatment as DFN might also reduce ferroptosis (iron-dependent apoptotic cell death) and other iron accumulation effects resulting in dysregulated cellular pathways after IVH. In addition, we endeavored to augment neuroprotection and to reduce cell death after IVH by mitigating inflammation with sulforaphane treatment (SFN; a naturally occurring plant Nrf2-antioxidant response element [ARE] activator that inhibits NFκB signaling). We found that after IVH, SFN-DFN treatment reduced the activation of M1 microglial phenotype plus reduced inflammation and cell death. We reasoned that a genome-wide RNAseq approach would unmask iron-specific toxicity signaling in brain sub-regions and microglial-related gene signatures. We delineated the transcriptomic changes associated with iron accumulation with DFN treatment and identified additional changes in iron pathway gene expression cascades.

## 2 Methods

### 2.1 Glycerol induced intraventricular hemorrhage in premature rabbit pups

Timed pregnant New Zealand White rabbits (*Oryctolagus cuniculus*) were obtained from Charles River Laboratories Inc. (Wilmington, MA, USA). Premature pups were delivered via cesarean section at day 29 (E29) of gestation (term = 32 days). Newborn premature pups were maintained and fed according to protocols outlined in our previous publications (Georgiadis et al., 2008; Chua et al., 2009; Vinukonda et al., 2010; Vinukonda et al., 2019).

Intraventricular hemorrhage (IVH) was induced by administering intra-peritoneal glycerol at 4–6 h of age, as previously described (Georgiadis et al., *2008*). The presence of IVH was confirmed by cranial ultrasound at 24 h of age. The severity of IVH was graded based on the echogenic volume within the ventricles, measured in three dimensions (length, width, depth) using both coronal and sagittal views. Hemorrhage was categorized into three grades: (1) no IVH: no detectable hemorrhage, (2) moderate IVH: clot volume of 100–250 mm^3^, with hemorrhage into the lateral ventricles and mild ventricular enlargement (distinct separation of the lateral ventricles), and (3) severe IVH: clot volume of 251–350 mm^3^, with marked ventricular enlargement (fusion of lateral ventricles into a single chamber) and/or parenchymal involvement.

### 2.2 Administration of pharmacological agents deferoxamine (DFN) and sulforaphane (SFN)

After ultrasound grading, pups were assigned to one of three experimental groups by matching birth weight and balanced with severity of IVH between treated and untreated IVH premature pups: (1) controls without IVH (balanced with equal number of no glycerol injected pups and glycerol injected but no IVH pups), (2) glycerol-IVH pups treated with saline, (3) glycerol-IVH pups treated with simultaneous SFN-DFN for all biochemical, cell density quantification and real time TaqMan gene expression assays. In the DFN monotherapy transcriptome analysis, we used a fourth group of pups with IVH that were treated only with DFN to identify iron regulated gene transcriptome changes vs. IVH alone. In this case, we sought to better understand which aspects of iron homeostasis or toxicity were altered by a DFN-dependent mechanism. We included 5 premature pups per experimental group for all endpoint studies except for tissue iron measurements for day 3 and day 7 where we used 6 pups for each experimental group.

We treated pups affected with IVH with deferoxamine (DFN; Hospira, INC. IL: 60045, Cat # NDC0409-2336-01) at 50 mg/kg (subcutaneously) beginning on day 1 for 3 days (3 doses; prior to sacrifice on day 3 postnatal age). A second group of IVH-pups received 5 doses, once daily for 5 days and were sacrificed on day 7. When using combined treatments, pups were also administered sulforaphane (SFN; EMD Millipore # 574215) dose, 25 mg/kg intramuscular (IM) at days 1 and 3 for day 3 endpoints, and days 1, 3 and 5 for day 7 endpoint studies. Both drug schedules are based on previous publications (Innamorato et al., 2009; Sripetchwandee et al., 2014; Cui et al., 2015; Klomparens and Ding, 2019).

For RNA sequencing tissue sample collection, we used the same DFN treatments as above for day 3 endpoint studies. The SVZ dissected tissue was collected following our previously published method (Finkel et al., 2023) and used for RNA isolation, sequencing, and transcriptome analysis. We used four samples in each experimental group for initial PCA analysis and in subsequent transcriptome data analysis. We excluded any outlier pups if their PCA values did not cluster with their cohorts.

### 2.3 Analgesia, anesthesia, and euthanasia of animals for tissue processing

#### 2.3.1 Preterm delivery

All experiments were performed according to American Veterinary Medicine Association (AVMA) guidelines and approved by the New York Medical College IACUC committee. During the non-survival cesarian section procedure performed on pregnant rabbits, deep anesthesia was induced using Isoflurane anesthesia by inhalation method (5%). Once deep anesthetic depth is achieved (vitals are monitored continuously and anesthetic depth is confirmed through lack of reaction to palpebral reflex and deep toe pinch), intra cardiac (I. C) injection of commercially available euthanasia solution (Somnasol, Med-Pharmex, Inc. CA; 1 mL/10 lb) is administered (euthanasia is confirmed by auscultating the heart). Extraction of live rabbit pups immediately follows the I. C. injection.

#### 2.3.2 Drug injections

We have injected Sulforaphane (intraperitoneal) and deferoxamine (intramuscular) using a 1 cc insulin syringe with a 29-gauge needle, under general anesthesia using inhalation of Isoflurane (1–2%) briefly for a few seconds. Post- administration of both medications, we observed no distress or evidence of pain. Animals are followed daily for dietary intake and weight gain (all pups are gavage fed) under supervision of the Department of Comparative Medicine Veterinary Staff.

#### 2.3.3 Harvesting tissues

Intraperitoneal (IP) injection of Ketamine (35 mg/kg) and Xylazine (5 mg/kg) was used for rabbit kits to induce sedation/anesthesia followed by euthanasia achieved through I. C. injection of commercially available euthanasia solution (1 mL/10 lb). All end-point experiments and collection of brain samples followed methods described in our previous publications and a schedule summarizing this schedule is included as Supplementary Table 3 (Vinukonda et al., 2010; Vinukonda et al., 2019; Finkel et al., 2023).

#### 2.3.4 Tissue processing after collection

All experimental procedures, sample collections, and laboratory assays were approved by the Institutional Animal Care and Use Committee (IACUC) at New York Medical College, Valhalla, NY. Forebrain parenchymal tissue, cerebrospinal fluid (CSF), and plasma samples were collected from all four experimental groups (control, IVH-saline, IVH + DFN, and IVH + SFN/DFN) at postnatal days 3 and 7. Each group included 5–6 pups per time point. CSF was collected via the anterior fontanelle, snap-frozen on dry ice, and stored at −80 °C for ELISA analysis. The subventricular zone (SVZ), including germinal matrix (GM), corpus callosum (CC), and corona radiata (CR), were manually dissected from 2–3 mm thick coronal brain sections (anterior to posterior midbrain) using a brain matrix slicer (Cat # BSRAS001-1, Zivic Instruments, Pittsburgh, USA), following our published methods for tissue dissection (Finkel et al., 2023). Samples were immediately snap-frozen in liquid nitrogen and stored at −80 °C for RNA extraction and ELISA or real-time PCR analysis. Forebrain tissue designated for immunohistochemistry (IHC) was fixed in 4% paraformaldehyde, embedded in optimal cutting temperature (OCT) compound, and coronally sectioned from 1.0 mm anterior to 1.0 mm posterior to bregma.

### 2.4 Histological evaluation of hematoma and iron accumulation

Hematoxylin and eosin (H&E) staining and iron staining were performed on fixed coronal forebrain sections using previously established protocols (Vinukonda et al., 2019). We assessed the distribution of hematoma and iron deposition in the SVZ in all experimental groups. Every 20th section was evaluated, resulting in approximately 10 serial sections per rabbit pup.

### 2.5 Quantification of iron deposition

SVZ tissue samples were lysed according to manufacturer methods. Protein concentration in the tissue lysates and CSF was quantified using the Pierce BCA Protein Assay Kit (Cat #23225, Thermo Scientific). The total iron content was determined using a colorimetric iron assay kit (Cat # ab83366, Abcam, USA). Quantification was performed according to kit protocol and manufacturer instructions.

### 2.6 Immunofluorescence staining (IHC)

Immunohistochemistry was performed as previously described (Vinukonda et al., 2010; Purohit et al., 2021; Finkel et al., 2023). Fixed forebrain coronal sections were rehydrated in 0.01 M PBS, incubated overnight at 4 °C with primary antibodies, followed by incubation with secondary antibodies at room temperature for 1 h. Sections were mounted using SlowFade™ Light Anti-fade Reagent (Molecular Probes, Invitrogen, CA, USA) and visualized under a fluorescence microscope. Primary antibodies used were: (1) Iba-1 (Cat # ab5076, Abcam) to detect total microglia, (2) Anti-Ki-67 (Cat # M724029-2, DAKO, Denmark) to assess cell proliferation, and (3) MHC class II/HLA-DR (Cat # M077501-2, Agilent/DAKO, Denmark) to identify M1 microglia. All sections were counterstained with DAPI. Sections were post-treated with an autofluorescence quencher TrueBlack® (catalog #23007, Biotium.com) to reduce background non-specific signal. TUNEL staining was performed to assess microglial apoptosis in combination with Iba-1 labeling.

### 2.7 Cell density and quantification procedures

Cell density quantifications, fluorescent images of the SVZ (including GM, CC, CR) were captured at high objective magnification (40x) after dual staining with Iba-1 (total microglia), MHCII/HLA-DR (M1 microglia) and data were shown scatter plot with bar graph. The fluorescence images for TUNEL (total apoptotic cells) and microglia cell death counts (Iba-1 combined with TUNEL double positive) were captured in 20x objective magnification. Two alternate serial sections per pup were analyzed and cell counts from each image of the 3 regions were summed and averaged for each animal, with 5 pups per group. TUNEL-positive cells were quantified following the manufacturer’s protocol (Apoptosis Kit Cat # 17–141, EMD Millipore/Sigma, St. Louis, MO, USA). Two investigators, blinded to group assignments, independently performed cell counts using ImageJ software with a grid overlay and their results were averaged. Data are presented as mean ± standard deviation (SD).

### 2.8 RNA isolation, RNA sequencing, and TaqMan assays for gene expression

Total RNA was extracted from SVZ dissected tissue using the Qiagen Total RNA Isolation Kit (Cat # 74104, Qiagen, USA). RNA quantity and integrity were assessed using a NanoDrop® Spectrophotometer ND-2000C (Thermo Fisher, Waltham, MA, USA). RNA sequencing was performed with ERCC (External RNA Control Consortium) spike-in controls added immediately after tissue collection for normalization and detection limit assessment. Libraries were prepared using the Illumina TruSeq Stranded mRNA Library Prep Kit and sequenced on the Illumina NextSeq-550 platform (paired-end, 2 × 75 bp) at the Genomics Core Laboratory at New York Medical College. The raw RNA-seq data are published and available in the GEO database (Accession # GSE185438 and GSE198497).

Gene expression and pathway analyses were conducted using the Partek Genomics Suite (ver.6.5) and Ingenuity Pathway Analysis system (Qiagen, CA, USA) respectively - after alignment and analysis of RNA-seq data. A false discovery rate (FDR) of 5% was applied to normalized data to identify differentially expressed genes (DEGs), which were used in IPA pipeline for network and pathway analysis as reported previously (PMID:22645653; 23958185). Differential gene expression and pathway/network analyses were performed in collaboration with the Genomics Core Facility at Rutgers New Jersey Medical School.

### 2.9 Statistical analysis

The rabbit pups were assigned randomly to the experimental groups. Scatter plots and bar graphs represent mean ± SD as described in the figure legends. The standard deviation (SD) was used to assess variation between individual of the samples in the group. TaqMan assays were performed in duplicate for each sample. Multiple groups were compared at individual postnatal ages using one-way ANOVA GraphPad Prism 6 (GraphPad Software, CA, USA) with a Tukey’s multiple comparison post-hoc analysis. A *p*-value of <0.05 was considered statistically significant.

## 3 Results

### 3.1 Simultaneous administration of sulforaphane (SFN) and deferoxamine (DFN) results in reduced hematoma and iron accumulation in the sub-ventricular region of developing brain after IVH in premature rabbit pups

**Figure 1:**
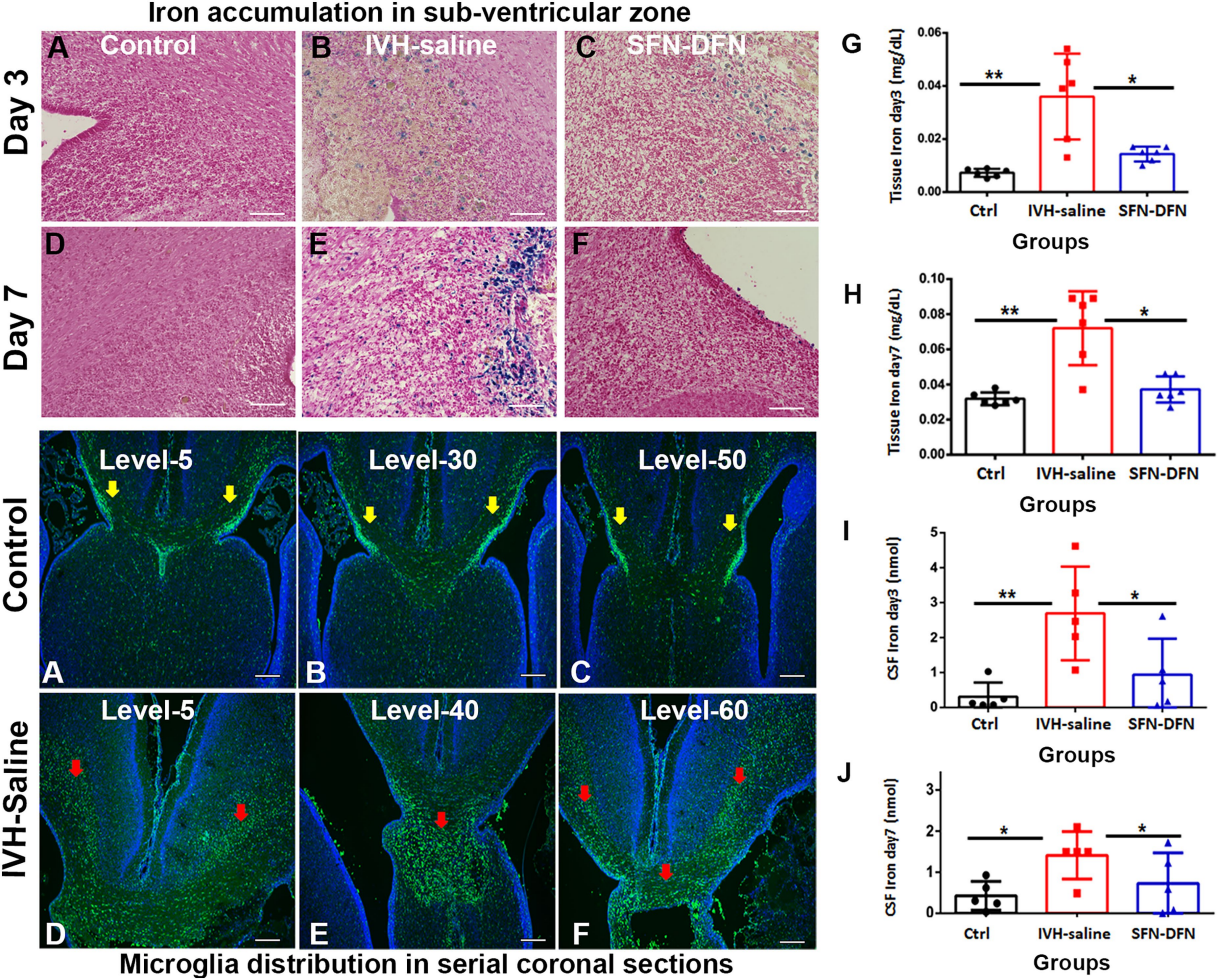
Hematoma/iron accumulation and distribution of microglia in subventricular zone (SVZ) in healthy controls and after intraventricular hemorrhage (IVH) in premature rabbit pups. [**(A–C)**, Upper Panel] Representative hematoxylin and eosin staining (H&E) on coronal section images showing hematoma and iron accumulation in the sub-ventricular regions of germinal matrix (GM) in healthy control and pups with IVH + saline at postnatal day 3. The images indicate hematoma (brown) and iron (blue) accumulation after IVH **(A vs. B)**. No hematoma or iron deposition was seen in healthy control at postnatal day 3 **(A)**. At postnatal day 3, IVH resulted in hematoma/iron accumulation that was reduced after SFN-DFN treatment **(C)**. **(D–F)** Representative hematoxylin and eosin staining (H&E) on coronal section images evaluating hematoma and iron accumulation in the sub-ventricular regions of the germinal matrix (GM) in healthy control (no IVH) and pups with IVH + saline at postnatal day 7. The images indicate hematoma (brown) and iron (blue) accumulation after IVH **(D vs. E)**. No hematoma or iron deposition was seen in healthy controls at postnatal day 3 **(D)**. The IVH resulted in hematoma/iron accumulation that was reduced after SFN-DFN treatment at postnatal day 7 **(F)**. [**(A–C)**, Lower Panel] Representative immunofluorescence-stained images on coronal serial sections using IBA-1^+^ antibody staining (total microglia marker) in the sub-ventricular zone and ventricular borders in healthy control pups at postnatal day 3. Developmentally dense distribution of microglial immunoreactivity was observed in the healthy controls. [**(D–F)**, Lower Panel] Representative immunofluorescence-stained images on coronal serial sections using IBA-1^+^ antibody staining (total microglia marker) counter stained with DAPI (nuclear marker) in the SVZ and ventricular borders in premature pups with and without IVH injury at postnatal day 3. Resident microglia primarily appeared as densely packed cells around the ventricle borders in healthy controls **(A–C)**. Whereas, after IVH + saline resident microglia infiltrated and dispersed away from the ventricle borders into deeper parenchymal areas **(D–F)** at early injury by postnatal day 3. The coronal serial sections of forebrain were taken from anterior to posterior regions and used for immunostaining (H&E and IHC). Images show multiple serial sections spaced 20–30 sections apart and matched anatomical regions in control and IVH pups. The images were taken using a Keyence microscope (Keyence Corporation of America, Illinois, USA). The scale bar for all images 20 μm. **(G–J)** Sulforaphane (SFN) and Deferoxamine (DFN) treatment diminished IVH- induced iron accumulation in tissue lysates and CSF during postnatal days 3 and 7 in premature rabbit pups. **(G,H)** Scatter plot bar graph shows SVZ dissected tissue lysates for free iron accumulation and clearance after SFN-DFN treatment in premature rabbit pups at postnatal day 3 **(G)** and for postnatal day 7 **(H)**. The data presented for tissue lysate as mg/dL with S. D. Sample size of 6 in each group for both day 3 and 7, *p < 0.05; **p < 0.01 were considered as significant. One way ANOVA applied for statistical comparisons. **(I,J)** Combined Sulforaphane (SFN) + Deferoxamine (DFN) treatment diminished IVH- induced iron accumulation in CSF during postnatal days 3 and 7 in premature rabbit pups. CSF collected from each experimental pup was used to assess iron levels. Total iron was assessed according to method described in the kit protocol. The data presented as nmols with S.D. Sample size of 5 in each group for both day 3 and 7, *p > 0.05, **p > 0.01 were considered as significant. One way ANOVA applied for statistical comparisons.

To assess whether IVH leads to hematoma and iron deposition in the germinal matrix (GM), subventricular zone (SVZ), and periventricular zone (PVZ), we conducted histological analyses using our well established glycerol-induced premature rabbit pup model of IVH (Georgiadis et al., 2008; Chua et al., 2009; Vinukonda et al., 2010; Vinukonda et al., 2019). The coronal forebrain tissue blocks were dissected along the anterior–posterior axis to include both lateral ventricles and the SVZ, following our published protocols and established methods for neurotoxicological evaluation in rabbits (Pardo et al., 2020; Purohit et al., 2021). As shown in Figures 1A–F, upper panel, (H&E), iron (blue) and hematoma (brown) deposits were observed in the GM and SVZ regions of IVH pups over postnatal day 3 (Figures 1A vs. B) and day 7 (Figures 1D vs. E). Whereas no such deposition was seen in healthy controls at either postnatal days. Of note the iron deposition increased by postnatal day 7 in IVH pups, suggesting progressive accumulation. Importantly, after SFN-DFN treatment this accumulation was suppressed at both postnatal ages (Figures 1B,E vs. C,F). The representative images shown in GM region of the SVZ for both postnatal ages days 3 and 7.

Further, there was a significant increase in free iron levels in the dissected SVZ tissue lysate after IVH compared with healthy control pups at day 3 (Figure 1G, *p* < 0.01, *n* = 6 in each group) and day 7 (Figure 1H, *p* < 0.01, *n* = 6 in each group); this deposition was significantly reduced after SFN-DFN treatment at both postnatal days 3 (Figure 1G, *p* < 0.05, *n* = 6 in each group) and day 7 (Figure 1H, *p* < 0.05, *n* = 6 in each group).

Similar changes were observed for iron quantification in CSF collected from the three experimental groups at postnatal day 3 and 7 (Figures 1I,J, *p* < 0.05, *n* = 5 in each group for both days 3 and 7). This data indicated that after IVH a significant accumulation of free iron occurred in the SVZ and that after SFN-DFN, this accumulation was reduced.

### 3.2 Microglia distribution around the lateral ventricles and effect of IVH on resident microglia at postnatal day 3

**Figure 2:**
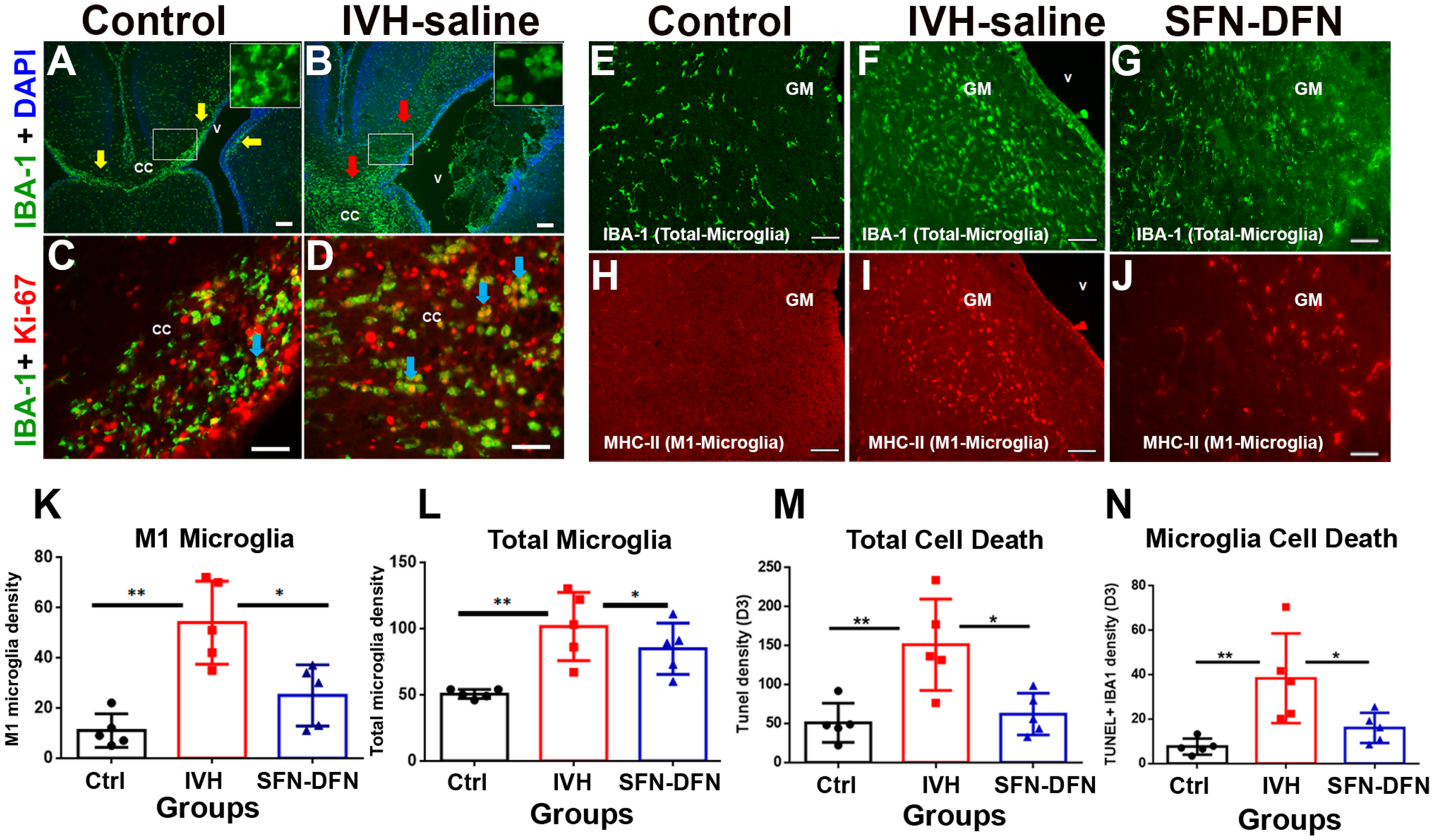
**(A–L)** Microglia distribution around the lateral ventricles and effect of IVH + saline on resident microglial phenotypic change and effects of SFN-DFN treatment on IVH in premature rabbit pups. **(A–D)** Representative immunofluorescence images showing microglia changes after IVH. **(A)** Iba-1 positive total microglia form dense tracks (immune signal in green) in the ventricular borders and corpus callosum (CC) in healthy controls [ramified morphology (inset-A), less proliferative and show few co-localized cells in yellow color **(C)**] at day 3. **(B)** After IVH, Iba-1^+^ cells now appear dispersed deeper into the parenchyma (SVZ and PVZ), show amoeboid morphology (inset-B), are highly proliferative shown as more co-localized dividing cells in yellow **(D)**. Yellow and red arrows show microglial tracks and dispersal deeper into the parenchyma **(A,B)**. Blue arrows show proliferating microglia, co-localized, Iba-1 with Ki-67 **(C,D)**. The low magnification images compare Iba-1^+^ immune signals for control and IVH **(A,B)** and at high magnification show Iba-1^+^ combined with Ki-67^+^ [proliferation marker; **(C,D)**] in premature pups at postnatal day 3. **(E–J)** Representative immunofluorescence staining on coronal sections showing total and activated M1 microglia and effect of SFN-DFN treatment at day 3. The coronal sections were stained with Iba-1 (total) and MHCII/HLA (M1 specific proinflammatory) antibodies. The Iba-1 positive (non-reactive, ramified) were few and no MHCII/HLA positive immune cells were seen in healthy controls **(E vs. H)**. After IVH + saline, a large number of microglia were positive for MHCII/HLA (red) indicating M1 microglia **(F vs. I)**. This M1 expansion was reduced after SFN-DFN treatment in IVH pups **(G vs. J)**. Scale bar 20 μm, Ventricle (v), corpus callosum (CC). **(K,L)** Scatter plot with bar graph, shows cell density quantification after SFN-DFN treatment suppressed both M1 infiltration and total microglia after IVH at postnatal day 3. Data shows significant increase in M1 [MHC-II positive, **(K)**] and total microglia [Iba-1^+^, **(I)**, right panel] cell density compared with healthy controls. This increased density was reduced after SFN-DFN treatment of IVH for both MI **(K)** and IBA-1 **(L)** at postnatal day 3. The cell count was performed on coronal section images taken in 40X magnification in both left and right hemispheres (counts include GM, CC and CR sub-regions). The data presented mean and SD in each group. Sample size of 5 pups in each experimental group, **p < 0.01, *p < 0.05 considered as significant. **(M,N)** Scatter plot with bar graph shows cell density quantification for total cell death (TUNEL positive cells) and specific microglia cell death (Iba-1^+^ + TUNEL double positive cells). The data indicated a significant increase of total and microglia specific cell death in IVH compared to controls at both postnatal ages day 3 and 7 **(J,K)**. Whereas after SFN-DFN treatment of IVH the total cell and microglial-specific cell death were suppressed at both postnatal days 3 and 7 **(J,K)**. The cell count was performed on 20X image. The data presented mean absolute cell count with SD. Sample size for TUNEL density and for dual stained TUNEL+IBA1^+^ cell density, each had 5 pups per group. *p < 0.05, **p < 0.05 considered as significant.

To assess the response to IVH of brain resident macrophage-microglia after free hemoglobin (fHb) and iron accumulation, we examined the density of Iba-1^+^ resident microglia in the CNS and the expansion/multiplication of microglial by cell division using Ki-67^+^ dual staining (Figures 1A–F lower panel, IF, serial sections and Figures 2A–D). In healthy controls, as expected, dense populations of Iba-1 positive microglia were observed along the ventricular borders, septal region (between the lateral ventricles), and choroid plexus versus lower microglial density in the white matter and cortex (Figures 1A,C, lower panel). On the other hand, after IVH, the densely populated microglial multiply several fold and are found further away from the ventricular borders deeper into the brain parenchyma (Figure 1D–F, lower panel, IHC, serial sections). On day 3, Iba-1 (Figures 2A,B) and dual staining with Iba-1 and Ki-67 antibodies identified total microglia population and those proliferating cells (Ki-67^+^) in coronal sections (Figures 2C,D). Healthy control pups exhibited a typical ramified microglial morphology with multiple processes (Figure 2A; inset, 40× high magnification) where few microglia were positive for Ki-67 immune reactivity, indicating minimal proliferation (Figure 2C; blue arrow). In contrast, following IVH, the infiltrated microglia acquired an amoeboid morphology and spread into deeper parenchymal regions (Figure 2B, inset, 40x high magnification). These infiltrated microglial cells were almost all positive for Ki-67 in IVH indicating strong mitotic activity (Figure 2D; blue arrows). These data indicate that after IVH, resident microglia are activated and become highly mitotic and appear to have migrated deeper into the parenchymal regions of the SVZ, CC and CR regions.

### 3.3 SFN-DFN treatment suppressed proinflammatory M1 microglia infiltration and cell death induced by IVH in the SVZ of the developing brain in premature pups

Immunofluorescent staining of total microglia (Iba-1^+^) and a M1 specific marker (MHC-II/HLA^+^) indicated that strong immune signal for total and activated microglia M1 phenotype in IVH compared to no IVH healthy controls at postnatal day 3 (Figures 2E,H, vs. F,I). And this immune reactivity was reduced after SFN-DFN treatment (Figures 2F,I vs. G,J). Further the cell density quantification for M1 and total microglia (Figures 2K,L) showed some effect of SFN-DFN on the rise in total microglia (Iba-1^+^) after IVH (Figure 2L); however, SFN-DFN specifically reduced the number of M1-phenotype proinflammatory microglia vs. IVH alone (MHC-II/HLA^+^; *p < 0.01, *n* = 5 each group). TUNEL staining for total cell death indicated strong immune signals in IVH pups compared with no IVH pups (Supplementary Figures 1A–C vs. D–F), whereas after SFN-DFN treatment the immune reactivity was reduced (Supplementary Figures 1D–F vs. G–I). Further, TUNEL positive cell death quantification assessment indicated a significant increase in total cell death in IVH compared with control which was decreased after SFN-DFN treatment compared with IVH alone at postnatal day 3 (Figure 2M; *p* < 0.01 and *p* < 0.05, *n* = 5 in each group for both comparisons). Similarly, dual staining TUNEL positive cells + Iba-1^+^ microglia showed significantly reduced microglia specific cell death after IVH when treated with SFN-DFN (Figure 2N; *p <* 0.01 and *p* < 0.05, *n* = 5 in each group for both comparisons).

### 3.4 Assessment of IVH induced transcriptome changes and effects of DFN treatment

To uncover global gene expression changes in IVH and to identify iron-regulated pathways affected by DFN treatment, we analyzed the transcriptomic profiles of SVZ dissected forebrain tissues. Our RNAseq raw data are publicly available in the GEO database (Accession # GSE185438 and GSE198497, published in 2022).

#### 3.4.1 Principal component analysis

**Figure 3:**
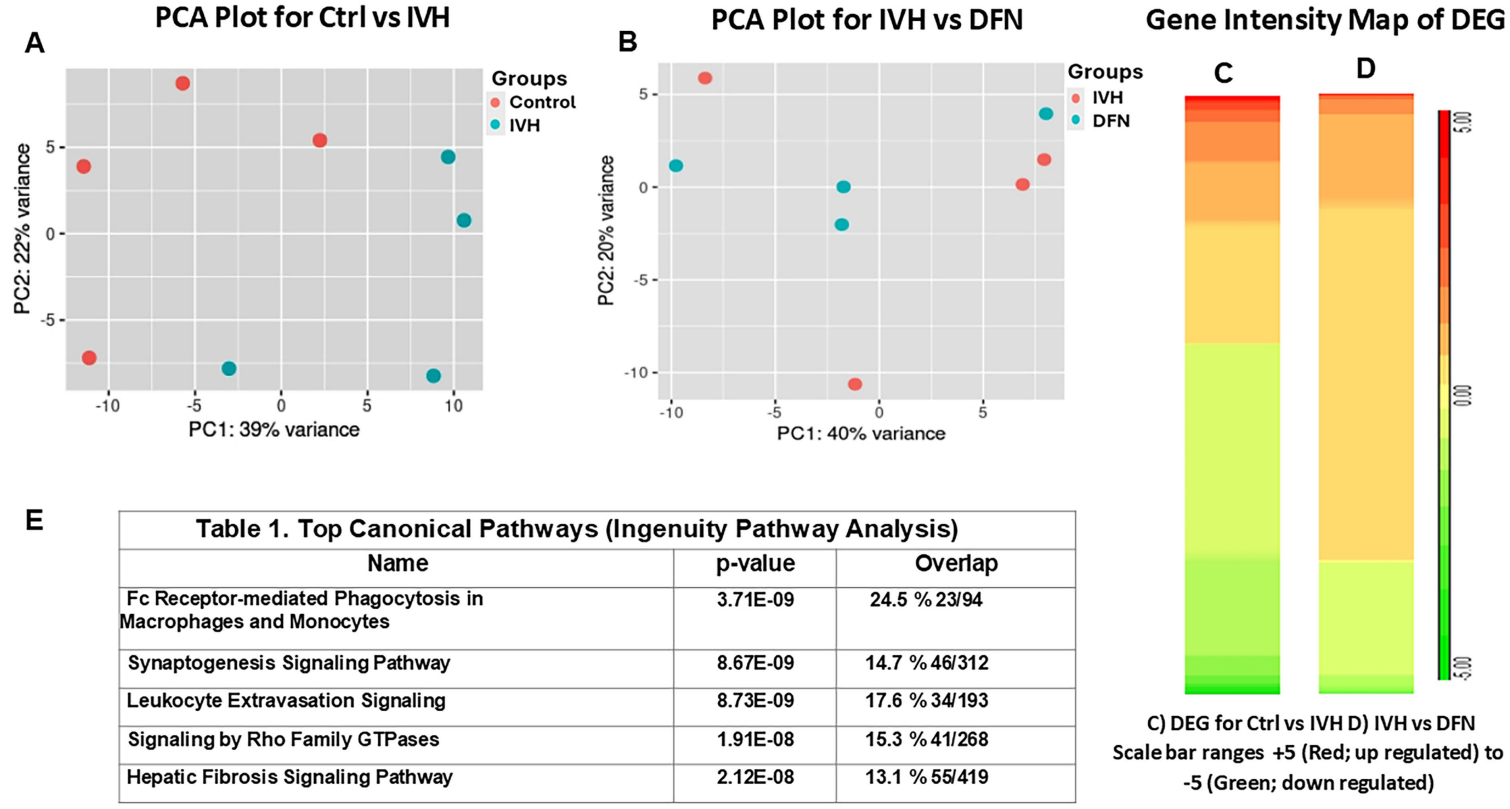
Genome-wide transcriptome changes in IVH + saline and the effect of IVH + DFN treatment on gene expression in dissected SVZ tissue at postnatal day 3 premature rabbit pups. **(A,B)** Principal component analysis (PCA) of RNAseq data highlights specific segregation and clustering of different experimental groups at postnatal day 3 (Ctrl: control, IVH; DFN-treated). The RNAseq data of samples (*n* = 4 per group) were distinctly distributed between healthy controls vs. IVH **(A)** and IVH + saline vs. IVH + DFN treated pups at day 3. The controls samples (red colored circles) formed a separate cluster independent of IVH (blue colored circles) based on their global gene expression profiles **(A)**. We do not see any overlay of red and blue circles gene expression profiles. The PCA plot between IVH vs. DFN treated samples also visualized separate clusters **(B)**. Note that in **(B)**, there was one outlier of each IVH and DFN data sets, which were excluded from further analysis (see main text). The label on X and Y axes show PC1 and PC2 variances, respectively. Total RNA isolated from SVZ dissected tissue was used for RNAseq. PCA analysis was performed as per standard pipeline processing of RNAseq data at the Genomics Core Laboratory at New York Medical College. **(C,D)** Intensity map of differentially expressed genes (DEG) in IVH + saline and after IVH + DFN treatment at postnatal day 3. Heat map of control vs. IVH + saline **(C)** indicated a total of 1,373 DEGs in IVH at postnatal day 3 and 440 DEGs when treated with DFN after IVH **(D)** treatment. Color intensity map in IVH visualized brighter (red intensity for upregulation and green for downregulation) with multiple blocks compared with controls **(C)**; both red and green color intensity was reduced after DFN treatment **(D)**. Data were derived from controls (*n* = 4), IVH + saline (*n* = 3), and IVH + DFN (*n* = 3) pups per group. The intensity of color in **(C,D)** is proportional to the degree of gene expression. Scale bar ranges from +5 (Red; upregulated) to −5 (Green; downregulated). Gene expression data presented in C and D corresponds to Log_2_ mean fold change. **(E)** Top canonical pathways identified by Ingenuity pathway analysis. Table showing the most significant canonical pathways across the entire data sets. The highest percentage of gene expression change related to phagocytosis function in macrophage and monocytes and leukocyte extravasation signaling in SVZ dissected RNA sequencing data at postnatal day 3. The significance indicates the probability of association of molecules in the study with the canonical pathway data sets in IPA, which utilizes Fishers Exact test for *p*-value calculations.

We first determined the distribution of variance in the RNAseq data of control versus IVH samples using principal component analysis (PCA). The PCA results represent the relative gene expression pattern of each sample, which demonstrates the biological variation of individual samples, even in the same group. In Figure 3A, control samples were clustered in the upper-left side (red dots), while IVH samples were clustered in the lower-right side (blue dots), which indicates the existence of a significant difference between the two groups. However, in Figure 3B, the samples in group IVH + saline vs. IVH + DFN were intermixed, where 3 of the 4 pups in each group clustered yet, one pup from each group was distinct from their cohorts (upper-left) and thus were excluded from subsequent analyses for DEG and canonical pathways.

#### 3.4.2 Analysis of differentially expressed gene intensity maps and top canonical pathways

The profile of significantly differentially expressed genes (DEGs) in the SVZ was visualized using heat intensity maps. IVH pups exhibited 1,373 DEGs compared with no IVH controls, while DFN treatment modulated 440 of these genes, compared to no treatment controls (Figures 3C,D). The gene intensity map and the number DEGs indicate that expression of about 30% of IVH dysregulated genes were impacted by DFN treatment. Further, the Ingenuity Pathway Analysis (IPA) of DEGs revealed several significantly dysregulated canonical pathways after IVH compared to controls, particularly in the innate immune signaling pathways such as Fcg-receptor-mediated phagocytosis and leukocyte extravasation cascades (Figure 3E, table). Moreover, 23 genes out of 94 in the phagocytosis pathway are related to microglia and macrophages (Supplementary Table 1). These findings highlight the inflammatory nature of IVH and the role of microglial phagocytic activity.

#### 3.4.3 Differentially regulated gene expression in IVH and the effect of DFN treatment

**Figure 4:**
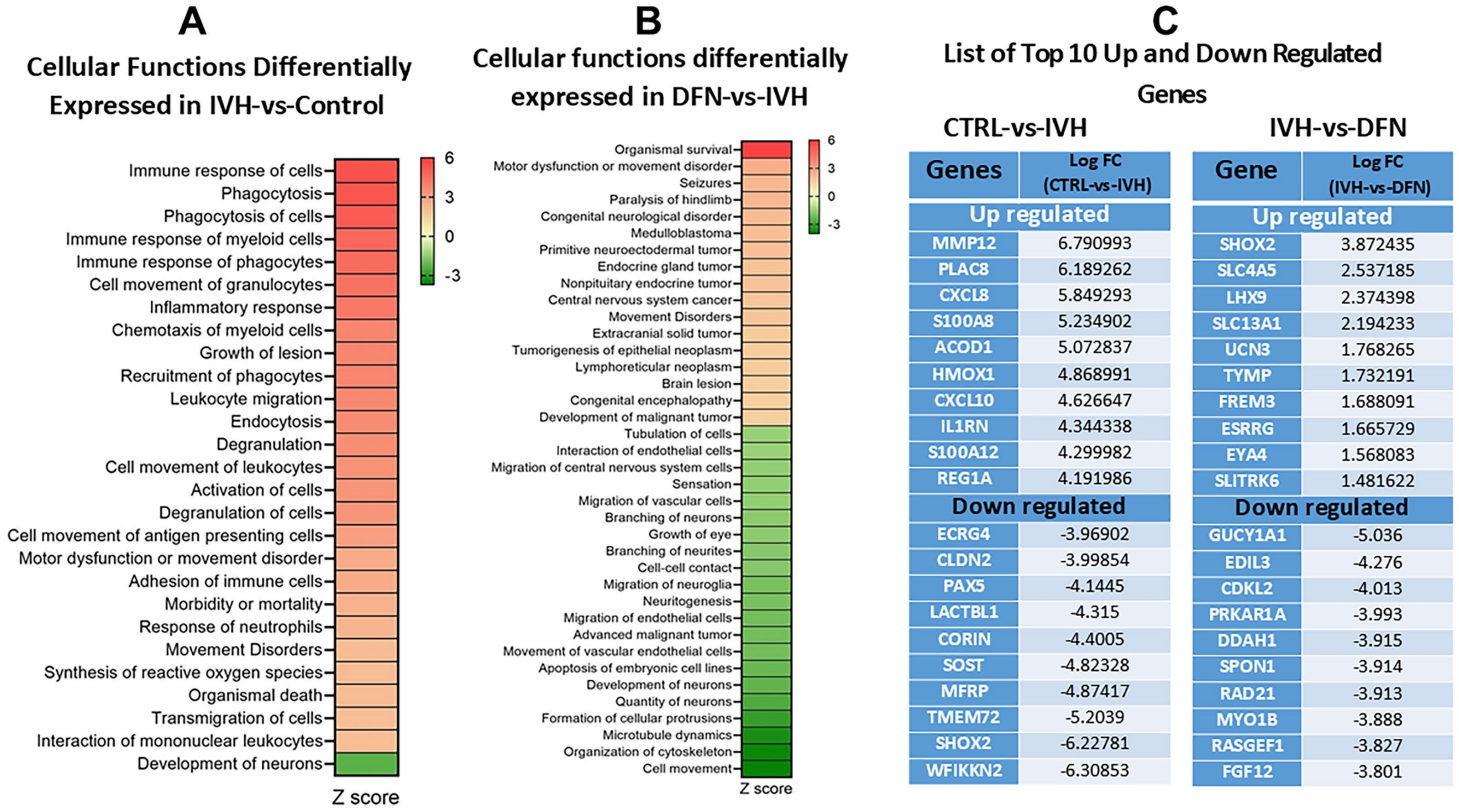
Differentially regulated cellular functions and list of highly up regulated/down regulated genes in IVH + saline and the effect of IVH + DFN treatment in premature rabbit pups. **(A,B)** Heat map of differentially regulated cellular biological functions in IVH + saline compared to controls [no bleed **(A)**] and in IVH + DFN treated versus no treatment groups **(B)**. IPA analysis of DEGs shows strong up regulation of cellular response of immunity, phagocytosis and inflammatory related genes in IVH, compared to controls **(A)**. However after IVH + DFN treatment many of the IVH upregulated responses were downregulated **(B)**. Color intensity indicates the magnitude of significance and the color scale is an indicator of significance based on z-scores. Red color indicates upregulation and green color indicates downregulation of cellular function. Data derived from *n* = 3–4 pups per group. Scale bar ranges from +6 (Red; upregulated) to −3 (Green; downregulated). **(C)** List of top 10 up- and downregulated DEGs in IVH + saline and effect of IVF + DFN treatment. The column on the left show’s gene symbols of up- and downregulated DEGs and their log2 fold change in IVH compared to control group (4-fold or higher is listed). The right column depicts up- and downregulated (Log2 fold change) DEGs in IVH vs. DFN treated groups at postnatal day 3. Data were derived from controls (*n* = 4), IVH + saline (*n* = 3) and IVH-DFN (*n* = 3) pups per group. The abbreviations of gene symbols shown in the table are expanded in Supplementary Table 2.

Based on our initial observation of significant changes of gene expression in IVH pups compared with healthy control pups, we sought to determine the cellular biological functional categories that were affected by the DEGs in the SVZ. As shown in Figure 4A, we observed upregulation of immune responses, particularly in innate cells, such as phagocytosis, inflammation and recruitment of activated leukocytes in IVH samples compared to controls (Figure 4A). Consistently, cellular functions associated with morbidity or mortality as well as tissue-level death were also upregulated in IVH. Thus, those cellular functions dysregulated in IVH are consistent with expected pathological sequelae. Next, we investigated the cellular biological functions affected by DFN treatment after IVH, compared to IVH + saline active controls. In the DFN-treated pups, cellular functions associated with activation and recruitment of immune cells were dampened (Figure 4B) and, consistently, cell survival function pathways were upregulated compared to the IVH + saline active controls. Furthermore, neuronal development pathways were also downregulated, reinforcing the importance of immune regulation interfering with normal brain development.

#### 3.4.4 Highly dysregulated genes in IVH and during DFN treatments

To further extract biological insights on specific genes that are involved with the pathophysiology during IVH, and after DFN treatment, we determined the top 10 most up- and downregulated DEGs and compared them between experimental groups (Figure 4C). The list of the top 10 upregulated genes in IVH + saline, compared to no IVH controls (all >4-fold) included: CXCL8, CXCL10, S100A8, S100A12 HMOX1, MMP12, PLAC8, ACOD1, IL1RN and REG1A. The down regulated genes in this group (ranging from 4- to 7-fold decrease) included: ECRG4, CLDN2, PAX5, LACTBL1, CORIN, SOST, MFRP, TMEM72, SHOX2 and WFIKKN2 (Figure 4C). The top 10 most significantly upregulated DEGs in IVH + DFN treated pups, include transcriptional regulators (SHOX2, LHX9, ESRRG, EYA4), neuronal signaling (UCN3, TYMP, FREM3) and ionic balance (SLC4A5, SLC13A1), while the downregulated DEGs are associated with endothelial function (EDIL3, SPON1, MYO1B), nitric oxide-cGMP/GTP signaling (GUCY1A1, DDAH1, PRKAR1A, RASGEF1, CDKL2), and regulation of organ development (RAD21, FGF12). In sum, the list of top up- and down-regulated genes in IVH (compared to no-IVH controls) and IVH + DFN treatment compared to IVH + saline active controls are consistent with their role in altering corresponding cellular functions shown in Figures 4A,B.

To confirm and validate some of the findings of RNAseq data on top 10 up- and downregulated genes in Figure 4C, we used TaqMan gene expression assays (Figures 6C–F) and we confirmed elevated expression of CXCL8, CXCL10 and S100 genes (S100A8 & S100A12) in IVH compared to controls. These over expressed chemokines are known to regulate their biological effects via CXCR1-3 receptor signaling (Zhou et al., 2023). S100A8 & A12 proteins also regulate TLR signaling mechanisms (Donato et al., 2013). Both types of ligands share MAP kinase (MAPK) signaling components to elicit distinct pathological effects on microglial activation to cause inflammation, apoptosis and other effector functions (Foell et al., 2007; Tao et al., 2022; Zhou et al., 2023).

#### 3.4.5 Identification of differently expressed canonical pathways and microglial network genes and effects of DFN treatment

**Figure 5:**
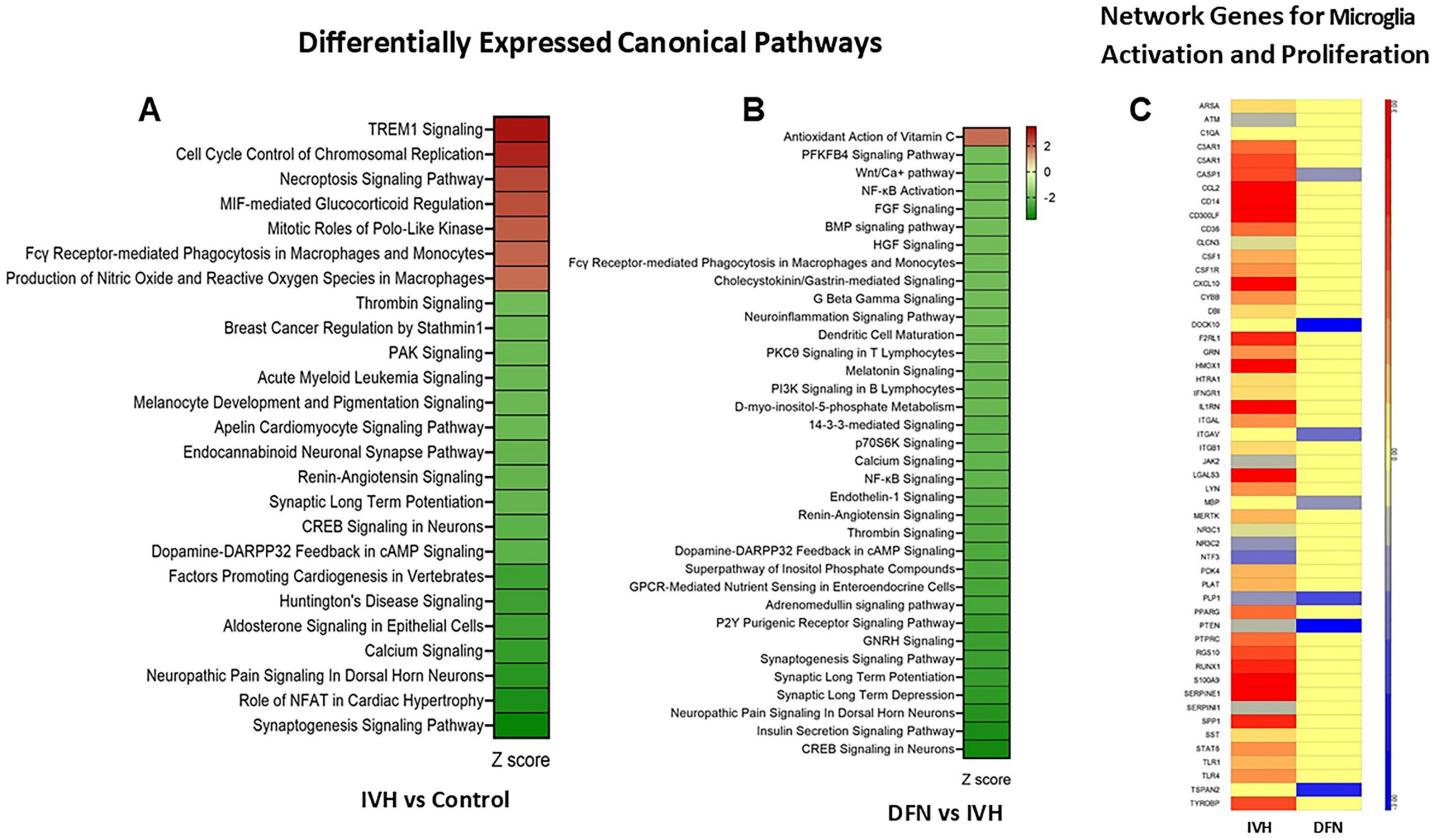
Differentially regulated canonical pathways and microglial activation and proliferation network gene expression in IVH + saline and the effect of IVH + DFN treatment. **(A,B)** Heat maps of differentially regulated canonical pathways in control vs. IVH + saline **(A)** or IVH + DFN treated versus IVH + saline **(B)** at postnatal day 3. Color intensity indicates the magnitude of significance and the color scale is an indicator of significance based on z-scores. Red color indicates upregulation and green color indicates downregulation of cellular function. Data derived from controls (*n* = 4), IVH + saline (*n* = 3) and IVH-DFN (*n* = 3) pups per group. Scale bar ranges from +2 (Red; upregulated) to −2 (Green; downregulated). **(C)** Heat map representing differentially expressed genes (DEGs) involved in microglia activation and proliferation in IVH + saline and treated with DFN at day 3. Left column for IVH + saline and right column for IVH + DFN treatment. Row indicates each gene as labeled and color-coded comparison between columns for each gene in row. Data derived from *n* = 3–4 pups per group. The intensity of color is proportional to the degree of gene expression. Scale bar ranges from +5 (Red; upregulated) to −5 (Blue; downregulated). Gene expression data corresponds to Log2 mean fold change. Data indicated large number of microglia activation and proliferation network genes over expressed in IVH but most of these genes were downregulated after DFN treatment. The abbreviations of gene symbols shown in the table are expanded in Supplementary Table 2.

To gain additional insights into the canonical pathways impacted by the DEGs of IVH and/or DFN treatment, we performed IPA Canonical pathway analysis. Our analysis revealed upregulated immune-related processes in IVH + saline vs. no IVH controls, including TREM1 signaling, MIF signaling, and reactive oxygen and nitric oxide production (Figure 5A). In contrast, neuronal pathways like cAMP, CREB, synaptogenesis, and thrombin signaling were down regulated. Importantly, IVH + DFN treatment down regulated many of the inflammatory response associated pathways including NFkB signaling, neuroinflammation signaling, Wnt/Ca pathway, P2YR signaling and CREB signaling in neurons (Figure 5B).

Gene network analysis highlighted that most of the affected genes in IVH are involved in microglia activation and participate by inducing inflammation, free radical production and cell death (Figure 5C). Consistently, microglial network genes involved in inflammation and cell death included: CASP1, DOCK10, ITGAV, TSPAN2, and PTEN that were upregulated in IVH, while the expression of each of these genes was downregulated after DFN administration (Figure 5C).

#### 3.4.6 Assessment of ferroptosis network genes expression in IVH and effect of DFN treatment

**Figure 6:**
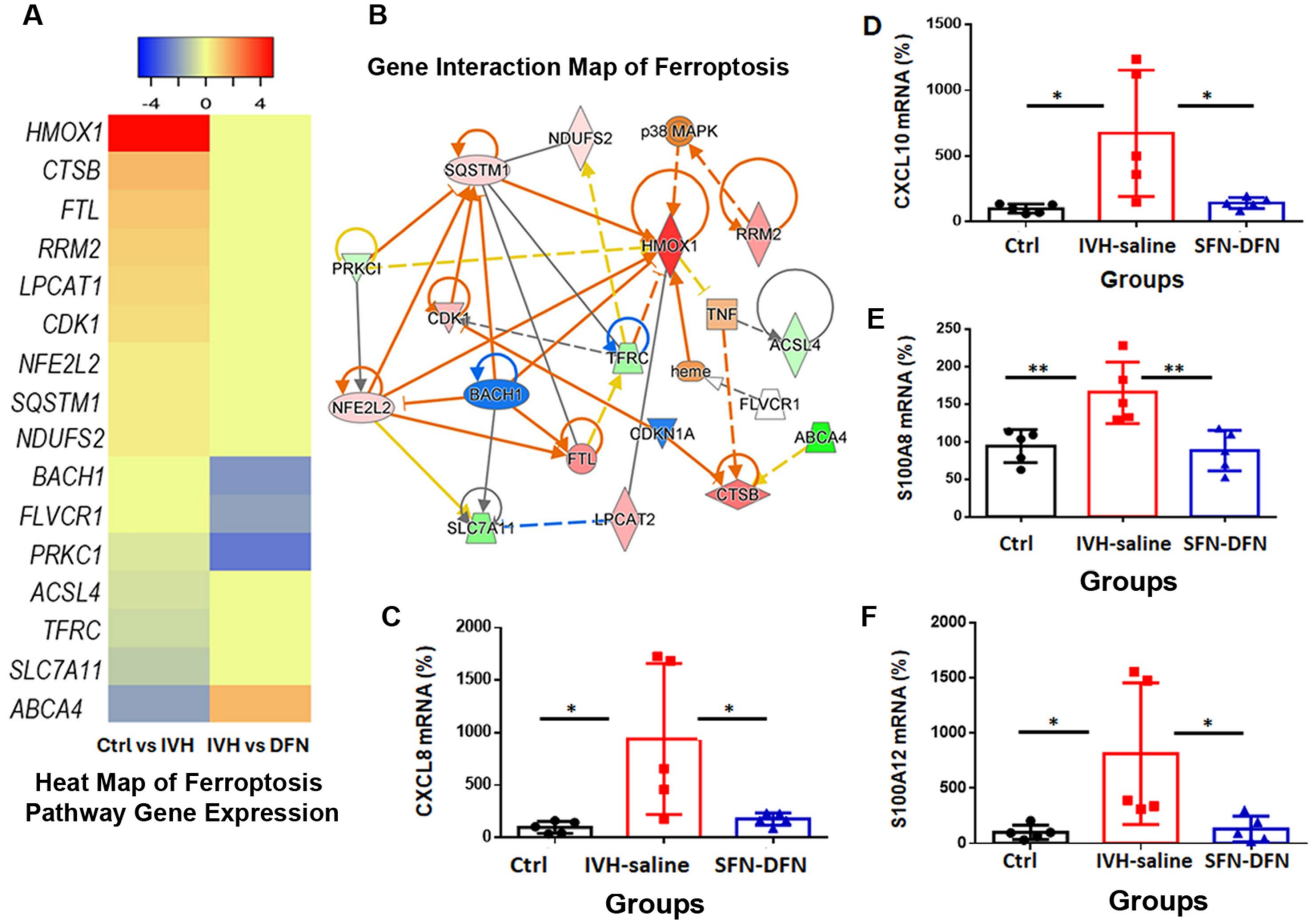
Heat map of ferroptosis network genes/pathway mechanisms and effect of DFN treatment and TaqMan assay confirmation of highly dysregulated genes in IVH and DFN treatment. **(A,B)** Heat map of ferroptosis pathway gene expression in IVH and treatment **(A)** and ferroptosis network interaction map **(B)**. The expression level and list of DEGs involved in ferroptosis network in IVH vs. Control (left column) and effects of DFN treatment, IVH vs. DFN (right column). Row indicates each gene as labeled and color-coded comparisons between columns for each gene in a row. Data derived from controls (*n* = 4), IVH + saline (*n* = 3) and IVH + DFN (*n* = 3) pups per group. The intensity of color is proportional to the degree of gene expression. Scale bar ranges from +4 (Red; upregulated) to −4 (Blue; downregulated). Gene expression data corresponds to Log2 mean fold change. Data indicated over expression of ferroptosis genes IVH + saline and this trend was suppressed after IVH + DFN treatment. **(B)** Image showing gene interactions among DEGs in ferroptosis networks. The color-coded symbols indicate functions of the transcript in interacting pathways. The intensity of color is proportional to the degree of gene expression. Red color indicates upregulation and green color indicates downregulation. Solid lines indicate direct interactions, and dotted lines indicate indirect interactions. The abbreviations of gene symbols shown in the table are expanded in Supplementary Table 2. Data indicated over expression of HMOX1 is a key molecule connected to P38 MAPK (Cell survival/death), while CDK1 and CSTB protease initiates cell death in IVH. Similarly, altered heme regulated pathway molecules like ACSL4, TFRC and FTL indicated iron homeostasis imbalance in IVH that was corrected to normalcy by DFN treatment. **(C–F)** SFN-DFN treatment suppressed selected inflammatory and chemokine gene expression that were induced by IVH + saline at postnatal day 3. The bar graphs show quantitative TaqMan gene expression using total RNA isolated from the SVZ of premature rabbit pups at day 3 and effects of IVH + SFN-DFN treatment. **(C,D)** The mRNA expression levels for chemokine CXCL8 **(C)** and CXCL10 **(D)** were significantly increased in IVH + saline compared with healthy controls and this increase was significantly reduced after IVH + SFN-DFN treatment at day 3. Sample size = 5 pups per group, *p < 0.05 for all comparisons. One way ANOVA applied for statistical comparisons. **(E,F)** The mRNA expression levels of calcium binding proteins S100A8 **(E)** andS100A12 **(F)** were significantly increased in IVH + saline compared with healthy controls and this increase was significantly reduced after IVH + SFN-DFN treatment at day 3. Sample size = 5 pups per group, *p < 0.05 (D&F); **p < 0.05 **(E)** for comparisons. One way ANOVA applied for statistical comparisons.

Iron toxicity causes cell death via mechanisms known as ferroptosis. We identified significant upregulation of a key marker of ferroptosis (HMOX1; Figure 5C). We further explored this in the form of heat maps to identify interacting partner genes involved in ferroptosis networks after IVH vs. no IVH controls and compared the effects of IVF + DFN treatment vs. IVH + saline active controls (Figure 6A, variable red color intensity). Key upregulated genes in the ferroptosis network in IVH + saline vs. control included: HMOX1, CTSB, FTL, PRM2, LPCAT1, and CDK1. In contrast, IVH + DFN treatment dampened these gene expressions, compared to the IVH + saline active control group. Importantly, expression of multiple key genes involved in iron metabolism and transport function included: ACSL4, TFRC, SLC7A11 and ABCA4 which were all downregulated in IVH + saline vs. no IVH controls and this trend was reversed in the IVH + DFN group compared to the IVH + saline treatment (Figure 6A). Further analysis of the interaction among ferroptosis-linked genes indicated that HMOX1 is the key molecule that connects several others, including p38 MAPK (cell survival/death) and ACSL4, TFRC, FTL (heme-iron regulating) pathways in IVH (Figures 6A,B). Related to this, up- regulation of CDK1 and CDKN1A would activate CTSB, a protease involved in initiating cell death and was observed in IVH pups. Importantly, expression of genes in this network were down regulated upon DFN treatment. Taken together, these findings underscore a role for DFN in modulating ferroptosis, particularly through HMOX1, and thus, possibly protecting developing brain tissue from ferroptosis after IVH. Future studies can address individual mechanisms using siRNA and knockout/knock-in *in vitro* and *in vivo.*

## 4 Discussion

It is well recognized that the recovery from perinatal IVH is complex and multifactorial involving a temporal dependence of both early and late injury mediators contributing to the disruption of postnatal brain development. In this report we first showed that a combination therapy of SFN-DFN after IVH can: (i) reduce free iron in the SVZ germinal zone and circulating CSF, (ii) mitigate the shift to inflammatory M1 microglia, (iii) reduce apoptosis. Moreover, DFN monotherapy (iv) mitigated the rise of microglial related proinflammatory genes and gene networks and (v) altered ferroptosis network genes and pathways as identified using RNAseq analysis sub-region analysis. This data suggests that early treatment with a pharmacologic intervention shortly after IVH can alter the course of injury and recovery by reducing the cascade of inflammatory responses arising from extravasation and lysis of red blood cells. It is plausible that these effects can lessen the hostile microenvironment during recovery from the injury incident and help recover processes of brain development. We speculate that these effects can extract a more favorable microenvironment effect for “*living cellular therapy*” to be effective when using exogenous stem cell interventions as these cells would also be less likely to die; effects on endogenous neuroprogenitor are yet to be determined (Vinukonda and La Gamma, 2022).

Initial histological analysis for hematoma and free iron accumulation showed that these deposits were significantly reduced after SFN–DFN combination treatment (Figure 1, upper panel, H&E). While under normal conditions, microglia densely populate the lateral ventricular borders in the subventricular zone (SVZ), and choroid plexus borders (Figure 1, bottom panel), we found that after IVH, microglia increased mitosis and cell density, thus appearing to have also migrated into the parenchyma. We did not determine whether circulating macrophages also contributed to this increase in microglial cell density. These microglia appeared to predominantly differentiate into the proinflammatory M1 phenotype; a sequence that was attenuated by SFN-DFN treatment (Figures 2E–J). Morphologically, these microglia exhibited an amoeboid shape and rounded cell bodies, consistent with a pro-inflammatory phenotype and a heightened activation state (Figures 2A–D; Colonna and Butovsky, 2017, Song and Colonna, 2018, Wang et al., 2023). During this critical developmental window, microglial activation is known to contribute to the release of pro-inflammatory cytokines and reactive oxygen species, exacerbating injury. Importantly, apoptotic microglia cell death (IBA-1 + TUNEL double positive cells) significantly increased after IVH (Supplementary Figure 1) yet, following SFN-DFN treatment, cell death was significantly reduced (Figures 2M,N). Taken together, our findings indicate that combined SFN-DFN treatment effectively reduces free iron accumulation, which was associated with fewer microglia transitioning to the M1 pro-inflammatory phenotype (Figures 2K,L).

Maintaining a balanced state between microglial activation, proliferation, and apoptosis is critical for normal postnatal brain development. Microglial proliferation and activation peaked on postnatal day 3 after IVH compared with no IVH. At that time, RNAseq data revealed significant alterations in gene expression profiles among IVH pups compared with healthy controls or in IVH compared with DFN monotherapy treated pups (at postnatal day 3). In the IVH group, 1,373 genes showed altered expression, while DFN treatment reduced that number to 440 differentially expressed genes, indicating a substantial modulatory effect. Notably, after IVH, among these changes were genes related to Fcγ-receptor-mediated phagocytosis in microglia (Figure 3).

Cell death can be influenced by factors such as lack of oxygen (e.g., due to mass compression effects or disruption of blood flow after IVH), glutamate excitotoxicity, or exposure to blood components like free iron. Our gene network analysis revealed that the top 10 up/down regulated genes involved upregulated transcripts (>4-fold) for key chemokines such as CXCL8 and CXCL10, along with damage-associated molecular pattern (DAMP) molecules S100A8 and S100A12 in IVH (Figures 4, 6). Elevation of genes involved in heme metabolism (e.g., HMOX1) and ferroptosis pathways further highlighted the complex, multifaceted nature of IVH pathology. Importantly, DFN treatment significantly downregulated the expression of these genes by postnatal day 3, suggesting early therapeutic efficacy and a potential window of opportunity as a therapeutic intervention.

Canonical pathway analysis identified activation of genes related to microglial functions, including inflammation, free radical generation, and cell death signaling. DFN monotreatment restored many of these pathways toward baseline expression levels (Figures 5A,B). Additionally, genes related to microglial proliferation and activation were markedly upregulated in IVH and normalized with DFN monotreatment (Figure 5C). Collectively, these findings support transcriptomic evidence of dysregulated innate immune and phagocytic responses to IVH where DFN treatment offers partial restoration of homeostatic gene expression patterns in microglia.

Our findings also indicated ferroptosis-related gene changes in the SVZ, driven by interactions among key regulators of ferroptosis, oxidative stress and inflammatory genes such as: HMOX1, TNF, CTSB, TFRC, FTL, and components of the NRF2/p38 MAPK pathway (Figure 6). These results suggest that iron removal alone may be necessary but is not sufficient to address the complex etiology of IVH-related injury and abnormal brain development.

Future implications of this work challenge the traditional focus on monotherapies targeting individual proinflammatory cytokines, growth factors, use of steroids, or single molecules in various pathways. Unfortunately, while instructive, former approaches yielded limited translational clinical successes (Wróblewska and Swietliński, 2005), **o**ne notable exception is the DRIFT protocol (Drainage, Irrigation, and Fibrinolytic Therapy). DRIFT demonstrated modest long-term benefits in neonates but was halted prematurely due to the risk of secondary perinatal hemorrhage (Whitelaw et al., 2010; Luyt et al., 2020). On the other hand, the significance of DRIFT findings underscored the importance of a multitiered approach directed at early interventions that concurrently address hematoma/iron clearance and attenuation of secondary inflammatory injury mechanisms arising from hemolysis-derived toxic mediators. In the current report, our results afford a new strategy using less invasive, pharmacologically driven tactical approaches (compared to DRIFT) to aid recovery from IVH.

Limitations of these experiments include the need for further validation of changes identified by RNAseq analysis using siRNA, gene knockout, over expression and single cell transcriptomic strategies to more specifically address effects on individual cell types. In addition, this proof-of-concept approach of transcriptome analysis needs independent quantification of the magnitude of RNA transcripts plus evidence that changes result in their cognate proteins. Also, experiments determining more precisely what proportion of the increase in activated microglial density arises from cell division, changes in phenotype or invasion of circulating macrophages would be of interest from both a biological and therapeutic perspective. Lastly, a more expanded analysis of drug dosing, timing, and combinatorial interactions is likely to be instructive on how best to exploit a combinatorial approach to benefit brain recovery during a time sensitive, severity and stage dependent, progression of complex pathophysiologic changes.

## 5 Conclusion

This study is the first to describe the early postnatal distribution of non-reactive brain-resident microglia densely populated along the borders of the lateral ventricles and choroid plexus in premature rabbit pups at early forebrain development on postnatal day 3. Following IVH, these microglia become activated, proliferate, and appear to migrate into deeper parenchymal regions of the injured brain. Notably, the activated microglia also exhibit major polarization into a proinflammatory M1 nomenclature phenotype that might result in a diminished proportion of the M2 protective phenotype - cells that are critical for tissue repair and resolution of inflammation and other developmental interactions in the developing brain. Importantly, SFN-DFN treatment mitigated M1 transformation, likely associated with reduced cell markers of inflammation and apoptotic cell death in the developing postnatal brain.

Furthermore, this study is the first to report in the context of IVH, dysregulation of the Fcγ receptor–mediated phagocytosis pathway, a key component of innate immune function in CNS microglia and macrophages. Transcriptomic analysis identified over expression of S100A8 and S100A12, pro-inflammatory calcium-binding proteins that serve as intracellular damage signals, as well as CXCL8 and CXCL10, potent chemokines expressed by neurons and microglia that act as key chemoattractants during neuroinflammation. Together, these findings offer insights into microglial dysfunction, innate immune network gene pathway dysregulation following IVH, and illustrate improvements after SFN-DFN combined treatment. What is clear is that strategically timed, combinatorial interventions are more likely to address specific stages of the injury pathways than monotherapies. *We speculate* that reduced inflammation will enhance the possibility of recovering normal brain maturation (Vinukonda et al., 2019) and that reducing endogenous cell death may also increase the likelihood of exogenous stem cell survival (“*living cellular therapy”*) plus stem cell effects while adapting to a changing local microenvironment (Vinukonda and La Gamma, 2022).

## Data Availability

The datasets presented in this study can be found in online repositories. The names of the repository/repositories and accession number(s) can be found in the article/Supplementary material.

## Supplementary material

SUPPLEMENTARY FIGURE 1 Intraventricular hemorrhage (+saline) results in enhanced TUNEL positive cell death, whereas after IVH + SFN-DFN administration TUNEL positive cells were reduced in the SVZ/GM at postnatal day 3. The coronal sections were stained using IBA-1^+^ (total microglia) and TUNEL (total cell death) or combined to identify microglial-specific apoptotic cell death. **(A–I)** Representative immunofluorescence staining images for coronal sections showing GM region of SVZ for IBA-1 positive total microglia **(A,D,G)**, TUNEL positive apoptotic cell death **(B,E,H)** and double positive cells for microglia specific cell death **(C,F,I)** for healthy controls, IVH + saline and IVH + SFN-DFN treatment, respectively, at day 3. The Iba-1, TUNEL and IBA-1 + TUNEL double immune positive cells were fewer in healthy controls compared with IVH-saline treated pups **(A–C vs. D–F)**, whereas this increase in IVH was reduced after SFN-DFN treatment **(D–F vs. G–I)** in IVH pups at postnatal day 3. All images were taken using a Keyence microscope (Keyence Corporation of America, Illinois, USA). The scale bar for all images 20 μm.
