# Supplementary figures and images for: Combination drug therapy reduces iron accumulation and microglia-mediated pathologies in neonatal intraventricular hemorrhage: a biochemical and transcriptomic analysis
Source: Front Cell Neurosci. 2026 May 25;20:1812529. doi: 10.3389/fncel.2026.1812529 (PMC13243052; doi:10.3389/fncel.2026.1812529)

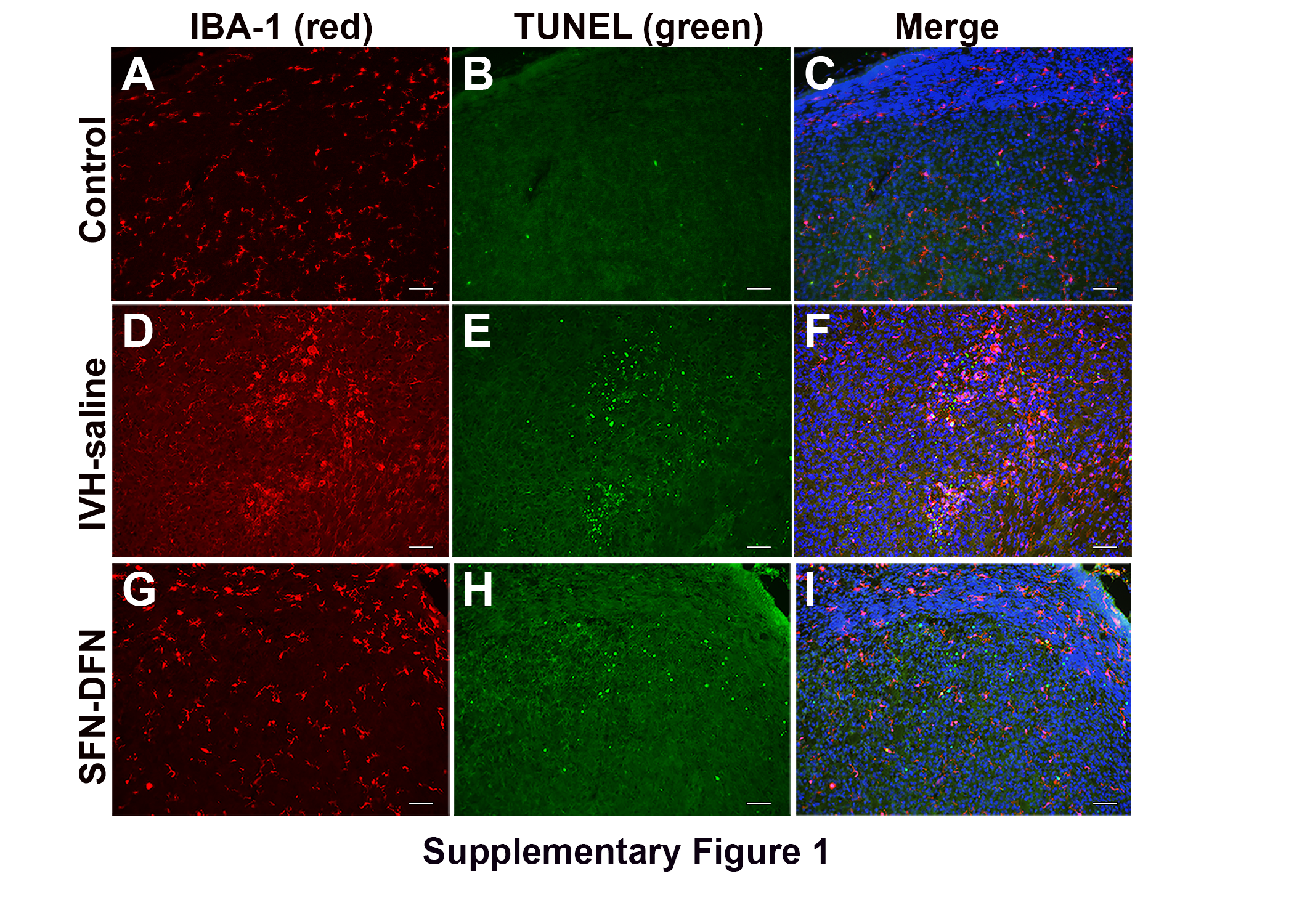

Supplement: SUPPLEMENTARY FIGURE 1 — Intraventricular hemorrhage (+saline) results in enhanced TUNEL positive cell death, whereas after IVH + SFN-DFN administration TUNEL positive cells were reduced in the SVZ/GM at postnatal day 3. The coronal sections were stained using IBA-1+ (total microglia) and TUNEL (total cell death) or combined to identify microglial-specific apoptotic cell death. (A–I) Representative immunofluorescence staining images for coronal sections showing GM region of SVZ for IBA-1 positive total microglia (A,D,G), TUNEL positive apoptotic cell death (B,E,H) and double positive cells for microglia specific cell death (C,F,I) for healthy controls, IVH + saline and IVH + SFN-DFN treatment, respectively, at day 3. The Iba-1, TUNEL and IBA-1 + TUNEL double immune positive cells were fewer in healthy controls compared with IVH-saline treated pups (A–C vs. D–F), whereas this increase in IVH was reduced after SFN-DFN treatment (D–F vs. G–I) in IVH pups at postnatal day 3. All images were taken using a Keyence microscope (Keyence Corporation of America, Illinois, USA). The scale bar for all images 20 μm. [file Supplementary_file_1.tif]
