# Supplementary material for: Combination drug therapy reduces iron accumulation and microglia-mediated pathologies in neonatal intraventricular hemorrhage: a biochemical and transcriptomic analysis
Source: Front Cell Neurosci. 2026 May 25;20:1812529. doi: 10.3389/fncel.2026.1812529 (PMC13243052; doi:10.3389/fncel.2026.1812529)
Supplement: Supplementary file 2 [file Table_1.DOCX]

| **Symbol** | **Entrez Gene Name** | **Expression Ratio**  **(IVH-vs-no IVH)** | |
| --- | --- | --- | --- |
| HMOX1 | Heme oxygenase 1 | 4.869 |  |
| RAC2 | Rac family small GTPase 2 | 2.296 |  |
| FYB1 | FYN binding protein 1 | 1.863 |  |
| SYK | Spleen associated tyrosine kinase | 1.688 |  |
| VAV1 | Vav guanine nucleotide exchange factor 1 | 1.588 |  |
| ARPC1B | Actin related protein 23 complex subunit 1B | 1.443 |  |
| LCP2 | Lymphocyte cytosolic protein 2 | 1.394 |  |
| INPP5D | Inositol polyphosphate-5-phosphatase D | 1.208 |  |
| HCK | HCK proto-oncogene, Src family tyrosine kinase | 1.103 |  |
| ACTA2 | Actin alpha 2, smooth muscle | 1.028 |  |
| LYN | LYN proto-oncogene, Src family tyrosine kinase | 0.811 |  |
| WAS | WASP actin nucleation promoting factor | 0.811 |  |
| PLD2 | Phospholipase D2 | 0.7 |  |
| VAMP3 | Vesicle associated membrane protein 3 | 0.585 |  |
| ACTC1 | Actin alpha cardiac muscle 1 | 0.429 |  |
| ARPC4 | Actin related protein 23 complex subunit 4 | 0.258 |  |
| PIK3R1 | Phosphoinositide-3-kinase regulatory subunit 1 | -0.308 |  |
| PRKCI | Protein kinase C iota | -0.514 |  |
| PLD5 | Phospholipase D family member 5 | -0.517 |  |
| PTEN | Phosphatase and tensin homolog | -0.586 |  |
| VAV3 | Vav guanine nucleotide exchange factor 3 | -0.753 |  |
| PRKCQ | Protein kinase C theta | -0.91 |  |
| EZR | Ezrin | -1.259 |  |

Supplementary Table 1. FcgR mediated phagocytosis in microglia and macrophages
