# Supplementary material for: Combination drug therapy reduces iron accumulation and microglia-mediated pathologies in neonatal intraventricular hemorrhage: a biochemical and transcriptomic analysis
Source: Front Cell Neurosci. 2026 May 25;20:1812529. doi: 10.3389/fncel.2026.1812529 (PMC13243052; doi:10.3389/fncel.2026.1812529)
Supplement: Supplementary file 3 [file Table_2.DOCX]

Supplementary Table 2. Expansion of Gene annotation used in the study

| **Gene Symbol** | **Gene Name** |
| --- | --- |
| MMP-12 | Matrix Metallopeptidase 12 |
| PLAC8 | Placenta-Associated 8 |
| CXCL8 | Chemokine (C-X-C motif) ligand 8 |
| S100A8 | S100 calcium-binding protein-A8 |
| ACDO1 | Aconitate decarboxylase-1 |
| HMOX1 | Heme oxygenase-1 |
| CXCL10 | Chemokine (C-X-C motif) ligand-10 |
| IL1RN | Interleukin-1 receptor antagonist |
| S100A12 | S100 calcium-binding protein A12 |
| REG1A | Regenerating Islet-derived 1- Alpha |
| ECRG4 | Esophageal cancer-related gene-4 |
| CLDN4 | Claudin-4 |
| PAX5 | Paired Box-5 |
| LACTB1 | Lactamase beta-like protein1 |
| CORIN | Corin |
| SOST | Sclerostin |
| MFRP | Membrane Frizzled-Related protein |
| TMEM72 | Transmembrane protein-72 |
| SHOX2 | Short Stature Homeobox-2 |
| WFIKKN2 | WAP, Follistatin/Kazal, Immunoglobulin, Kunitz And Netrin Domain Containing 2 |
| SLC4A5 | Solute Carrier Family 4 Member 5 |
| LHX9 | LIM Homeobox Protein 9 |
| SLC13A1 | Solute Carrier Family 13 Member 1 |
| UCN3 | Urocortin-3 |
| TYMP | Thymidine Phosphorylase |
| FREM3 | FRAS1 Related Extracellular Matrix 3 |
| ESRRG | Estrogen Related Receptor Gamma |
| EYA4 | Eyes Absent Homolog 4 |
| SLITRK6 | SLIT and NTRK-like family member 6 |
| GUCY1A1 | Guanylate Cyclase 1 Soluble Subunit Alpha 1 |
| EDIL3 | EGF-like repeats and discoidin I-like domains 3 |
| CDKL2 | Cyclin Dependent Kinase Like 2 |
| PRKAR1A | Protein Kinase CAMP-Dependent Regulatory Type I Alpha |
| DDAH1 | Dimethylarginine Dimethylaminohydrolase 1 |
| SPON1 | Spondin-1 or F-spondin |
| RAD21 | RAD21 Cohesin Complex Component |
| MYO1B | Myosin 1B |
| RASGEF1 | Ras-Guanine Exchange Factor 1A |
| FGF12 | Fibroblast Growth Factor 12 |
| ARSA | Arylsulfatase A |
| ATM | Ataxia-Telangiectasia Mutated |
| C1QA | Complement C1q A Chain |
| C3AR1 | Complement Component 3a Receptor 1 |
| C5AR1 | Complement Component 5a Receptor 1 |
| CASP1 | Caspase-1 |
| CCL2 | Chemoattractant Protein-1 |
| CD14 | Cluster of Differentiation 14 |
| CD300LF | CD300 Molecule-Like Family Member F |
| CD36 | Cluster of Differentiation 14 |
| CLCN3 | Chloride Voltage-Gated Channel 3 |
| CSF1 | Colony-Stimulating Factor 1 or Macrophage Colony-Stimulating Facto (M-CSF) |
| CSF1R | Colony-Stimulating Factor 1 Receptor |
| CYBB | Cytochrome b-245, Beta Chain |
| DBI | Diazepam Binding Inhibitor, Acyl-CoA Binding Protein |
| DOCK10 | Dedicator of Cytokinesis 10 |
| F2RL1 | Coagulation Factor II Thrombin Receptor-Like 1 |
| GRN | Granulin Precursor |
| HTRA1 | High Temperature Requirement A Serine Peptidase 1 |
| IFNGR1 | Interferon Gamma Receptor 1 |
| ITGAL | Integrin Subunit Alpha L |
| ITGAV | Integrin Subunit Alpha V |
| ITGB1 | Integrin subunit Beta 1 |
| JAK2 | Janus kinase 2 |
| LGALS3 | Galectin-3 |
| LYN | LYN Proto-Oncogene, Src Family Tyrosine Kinase |
| MBP | Myelin Basic Protein |
| MERTK | MER Proto-Oncogene, Tyrosine Kinase |
| NR3C1 | Nuclear Receptor Subfamily 3, Group C, Member 1 |
| NR3C2 | Nuclear Receptor Subfamily 3 Group C, Member 2 |
| NTF3 | Neurotrophin 3 |
| PDK4 | Pyruvate Dehydrogenase Kinase 4 |
| PLAT | Plasminogen Activator, Tissue Type |
| PLP1 | Proteolipid Protein 1 |
| PPARG | Peroxisome Proliferator-Activated Receptor Gamma |
| PTEN | Phosphatase and Tensin Homolog |
| PTPRC | Protein Tyrosine Phosphatase, Receptor Type C |
| RGS10 | Regulator of G-protein Signaling 10 |
| RUNX1 | RUNX Family Transcription Factor 1 |
| S100A9 | S100 calcium-binding protein A9 |
| SERPINE1 | Serpin Family E Member 1 |
| SERPIN1 | Serine Proteinase Inhibitor 1 |
| SPP1 | Secreted Phosphoprotein 1 |
| SST | Somatostatin |
| STAT6 | Signal Transducer and Activator of Transcription 6 |
| TLR1 | Toll Like Receptor 1 |
| TLR4 | Toll Like Receptor 4 |
| TSPAN2 | Tetraspanin 2 |
| TYROBP | Transmembrane Immune Signaling Adaptor |
| CTSB | Cathepsin B |
| FTL | Ferritin Light Chain |
| RRM2 | Ribonucleotide Reductase Regulatory Subunit M2 |
| LPCAT1 | Lysophosphatidylcholine Acyltransferase 1 |
| CDK1 | Cyclin Dependent Kinase 1 |
| NFE2L2/NRF2 | Nuclear Factor, Erythroid 2-Like 2, code NRF2 protein |
| SQSTM1 | Sequestosome 1 |
| NDUFS2 | Ubiquinone Oxidoreductase Core Subunit S2 |
| BACH1 | Transcription factor BTB and CNC homology 1 (Bach1) |
| FLVCR1 | Feline Leukemia Virus Subgroup C Receptor 1 |
| PRKC1 | Protein Kinase C Iota |
| TFRC | Transferrin Receptor |
| SLC7A11 | Solute Carrier Family 7 Member 11 |
| ACSL4 | Acyl-CoA Synthetase Long-Chain Family Member 4 |
| ABCA4 | ATP Binding Cassette Subfamily A Member 4 |
|  |  |
