# Supplementary material for: Combination drug therapy reduces iron accumulation and microglia-mediated pathologies in neonatal intraventricular hemorrhage: a biochemical and transcriptomic analysis
Source: Front Cell Neurosci. 2026 May 25;20:1812529. doi: 10.3389/fncel.2026.1812529 (PMC13243052; doi:10.3389/fncel.2026.1812529)
Supplement: Supplementary file 4 [file Table_3.DOCX]

| **Experimental Assay** | **Sample collection Experimental type** | **Postnatal age and Sample size** |
| --- | --- | --- |
| [Hematoxylin and Eosin (H&E) staining](https://www.google.com/search?q=Hematoxylin+and+Eosin+%28H%26E%29+staining&client=safari&hs=5BzU&sca_esv=c886fa4b6d8c3132&channel=mac_bm&source=hp&ei=eWXSaaWSM9WnptQPrLLLwA8&iflsig=AFdpzrgAAAAAadJzid_THNpoub2y8F0fcUPl8cvpyVDK&ved=2ahUKEwili-Ov6taTAxW8z_ACHQ4tKrMQgK4QegYIAQgAEAM&uact=5&oq=H%26+e+staining&gs_lp=Egdnd3Mtd2l6Ig1IJiBlIHN0YWluaW5nMgkQABiABBgKGAsyCRAAGIAEGAoYCzIJEAAYgAQYChgLMgkQABiABBgKGAsyCRAAGIAEGAoYCzIJEAAYgAQYChgLMgkQABiABBgKGAsyCRAAGIAEGAoYCzIJEAAYgAQYChgLMgkQABiABBgKGAtI50ZQAFidRHAAeACQAQCYAVqgAekFqgECMTO4AQPIAQD4AQGYAg2gAsQGwgIREC4YgAQYsQMYgwEYxwEY0QPCAgUQLhiABMICDhAuGIAEGLEDGMcBGNEDwgIIEAAYgAQYsQPCAgsQLhiABBjHARjRA8ICDhAAGIAEGIoFGLEDGIMBwgIOEC4YxwEYsQMY0QMYgATCAgsQABiABBixAxiDAcICBRAAGIAEwgINEC4YgAQYxwEY0QMYCsICBxAAGIAEGArCAgoQABiABBjJAxgKwgIKEAAYgAQYkgMYCsICCxAAGIAEGIoFGJIDwgIGEAAYFhgemAMAkgcCMTOgB5Z4sgcCMTO4B8QGwgcIMC4xLjExLjHIBzyACAE&sclient=gws-wiz&sei=hGXSaaWOMI-Ew8cP4uG3oAk) | Fixed forebrain coronal tissue block sections. For assessment of iron and fHb in GM/SVZ, CC and CR regions of the forebrain parenchyma. | Postnatal Days 3 and 7. A sample size of 5 premature pups were included in each experimental group. |
| Iron quantification | Cerebrospinal fluid (CSF) and dissected SVZ tissue lysate. | Postnatal Day 3. A sample size of 6 premature pups tissue and CSF were included in each experimental group. |
| Immunofluorescence staining | Fixed forebrain coronal tissue block sections were used for assessment of microglia cell density, proliferation of microglia and cell death imaging and cell density quantification assessment. 40X objective image field was used for cell density.  IBA-1(Total), MHCII/HLA (M1) assessed in GM/SVZ, CC and CR regions of the forebrain parenchyma.  TUNEL and IBA1-TUNEL assessed in the GM/SVZ, CC regions of the forebrain parenchyma. 20X image field was used for mean cell density. | Postnatal Day 3. A sample size of 5 premature pups tissue block sections were included in each experimental group. |
| RNA sequencing | Forebrain SVZ parenchymal tissue dissected (Finkel et al, 2023). | Postnatal Day 3. |
| Note: Intraperitoneal (IP) injection of Ketamine and Xylazine used for anesthesia and euthanasia was achieved through I.C. injection of euthanasia solution for all experimental end points and samples collection. | | |
| Reference: Dina A Finkel, Ana Malfa, et al 2023, Stem Cells Translational Medicine, Volume 12, Issue 12, December 2023, Pages 811–824. | | |

**Supplementary Table 3: Experimental Assays and Sample collection methods**
